# Left atrial remodeling and voltage-guided ablation outcome in persistent atrial fibrillation patients over 75 years of age

**DOI:** 10.1016/j.hroo.2024.12.006

**Published:** 2024-12-24

**Authors:** Halim Marzak, Clément Baldacini, François Severac, Simon Fitouchi, Thomas Cardi, Mohamad Kanso, Alexandre Schatz, Patrick Ohlmann, Olivier Morel, Laurence Jesel

**Affiliations:** 1 Division of Cardiovascular Medicine, Nouvel Hôpital Civil, Strasbourg University Hospital, Strasbourg, France; 2 Public Health Service, Groupe Méthodes en Recherche Clinique (GMRC), Strasbourg University Hospital, Strasbourg, France; 3 UR 3074 Translational CardioVascular Medicine CRBS, Strasbourg, France

**Keywords:** Atrial fibrillation, Atrial remodeling, Catheter ablation, Elderly patients, Low-voltage zones, Voltage-guided ablation, Voltage map

## Abstract

**Background:**

The prevalence of atrial fibrillation (AF) increases with age. The improvement in ablation techniques has widened the indications, particularly in elderly patients. Data on LA remodeling and low-voltage zone (LVZ) extent in this subgroup are scarce.

**Objective:**

We assessed the left atrial (LA) bipolar voltage, LVZ extent, and efficacy of voltage-guided ablation in a cohort of patients with persistent AF according to age.

**Methods:**

Three hundred fifty-three patients with persistent AF undergoing a first voltage-guided ablation procedure were enrolled and divided into 2 groups: those <75 years of age (n=286) and those ≥75 years of age (n=67). LA voltage maps were obtained in sinus rhythm. *LVZ* was defined as <0.5 mV. A propensity score–matching analysis was used to assess the impact of age on LA remodeling.

**Results:**

The LA bipolar voltage was lower (*P*<.01) in elderly patients. LVZs were found in 67% of elderly patients and 30% of younger patients (*P*<.01), especially in mild (*P*<.01) and moderate (*P*<.01) LVZs. After propensity score matching, these differences were no longer noticeable. Pulmonary vein isolation alone was performed in 33% of elderly patients and 70% of patients <75 years of age (*P*<.01). Female sex (*P*<.001), age ≥ 75 years (*P*=.042), estimated glomerular filtration rate (*P*=.009), and LA volume index (*P*<.001) were predictive of LVZ presence. After 36 months of follow-up, the AF-free survival rate after a single procedure was similar between the 2 groups.

**Conclusion:**

Patients >75 years of age with persistent AF display increased LA substrate remodeling than do younger patients. LA scar did not seem to negatively affect the results of substrate-guided ablation, and the complication rate was low.

**Key Findings**

▪ In persistent atrial fibrillation (AF), elderly patients display more left atrial (LA) electrophysiological remodeling with lower bipolar voltage, more low-voltage zones, and higher LA volume.
▪ After matching the cohort according to cardiovascular risk factors, these differences are not so clear, testifying to the complex interplay of all cardiovascular risk factors in addition to age in the occurrence of LA remodeling.
▪ Despite this significant fibrotic remodeling, they have a similar and favorable 36-month outcome after a single voltage-guided ablation procedure with low complication rates.
▪ In our analysis, female sex, age ≥75 year, renal function, and LA indexed volume were predictors of the low-voltage zone in patients with persistent AF.
▪ No predictive factors for atrial arrythmia recurrence could be identified in this whole cohort.

## Introduction

Atrial fibrillation (AF) in the elderly population has become one of the most important public health problems with a considerable impact on health care systems.^1^ AF is common in elderly people. Its prevalence is strongly associated with increasing age.^2^

Elderly patients display more often cardiovascular risk factors such as hypertension and type 2 diabetes contributing to a higher risk of stroke. Chronic renal insufficiency and heart failure are also common in this subgroup of patients.^1^

The high number of comorbidities in the elderly population strongly limits the use of antiarrhythmic therapy, resulting in an increased risk of adverse effects due to drug intolerances and drug-drug interactions.^3^

Therefore, catheter ablation (CA) could represent a definitive solution for maintaining sinus rhythm (SR) in the elderly population. Patients ≥75 years of age could have a better maintenance in SR with CA than with antiarrhythmic drugs (AADs).^4^ Thus, CA has become an increasingly important part over this last decade in elderly patients^5^^,^^6^ while pharmacological rate control with atrioventricular node ablation was still a preferred option in this population before the ablation era.

The presence of low-voltage zones (LVZs) is well-known to be a powerful predictor of recurrence after CA.^7^ The association between LVZ and age has been reported in several studies.^8^^,^^9^ Data on the LVZ extent and its regional distribution in elderly patients (≥75 years of age) are currently scarce.^10^

LVZ-guided ablation in addition to pulmonary vein isolation (PVI), a tailored approach for persistent AF CA, could be more efficient in maintaining SR compared with other strategies.^11^, ^12^, ^13^, ^14^

The goals of this study were to assess both in an unmatched and in a matched cohort of patients with persistent AF referred for CA the left atrial (LA) bipolar voltage amplitude, LVZ extent, and its regional distribution and to compare data between patients ≥75 years of age and those <75 years of age.

The predictive factors for LVZ occurrence and the results of LA voltage–guided substrate ablation were also assessed.

## Methods

### Study population

**Figure 1:**
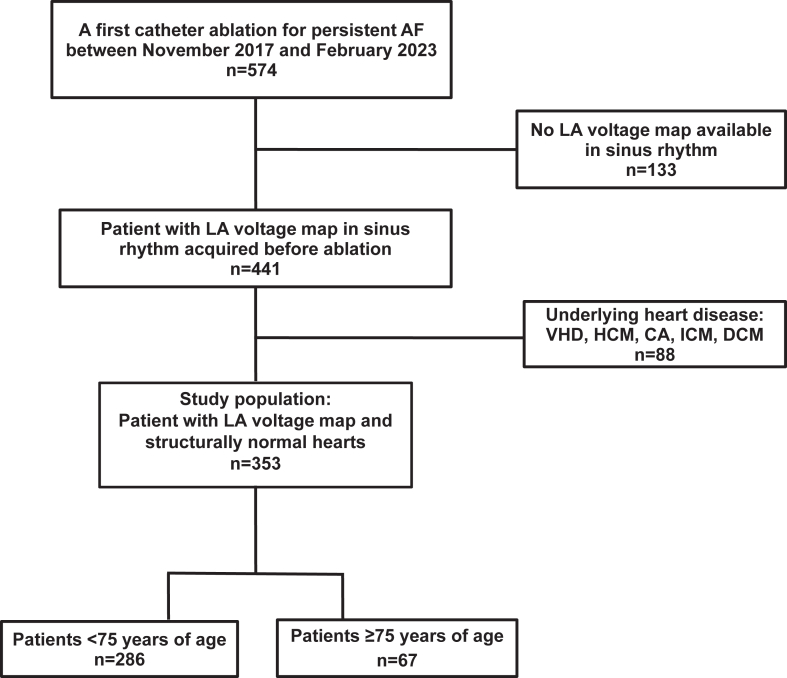
Flowchart of the study. Of the 574 patients admitted for initial radiofrequency ablation of persistent AF between November 2017 and February 2023, 353 (62%) met the inclusion criteria. One hundred thirty-three patients (23%) had no LA voltage mapping in sinus rhythm, and 88 (15%) were excluded from the analysis because of structural heart disease. AF = atrial fibrillation; CA = cardiac amyloidosis; DCM = dilated cardiomyopathy; HCM = hypertrophic cardiomyopathy; ICM = ischemic cardiomyopathy; LA = left atrial; VHD = valvular heart disease.

Five hundred seventy-four patients underwent initial ablation of persistent AF at our center between November 2017 and February 2023. After exclusion of 133 patients (23%) without LA voltage maps in SR and of 88 patients (15%) with structural heart disease (Figure 1), a total of 353 patients were finally enrolled in this observational study. The unmatched cohort was divided into 2 groups: those <75 years of age (n=286) and those ≥75 years of age (n=67). *Structural heart disease* was defined as ischemic heart disease, valve dysfunction (moderate, severe and massive), or primary myocardial structural disease including dilated cardiomyopathy and hypertrophic cardiomyopathy. Patient demographic and baseline clinical characteristics were recorded.

This study protocol adhered to the Declaration of Helsinki and was approved by the Institutional Review Board of the University of Strasbourg (CE-2023-113). All patients provided written informed consent.

### Propensity score–matched cohort study

We used an inverse probability of treatment weighting approach based on the propensity score (PS) to balance the baseline characteristics between the 2 groups.^15^ The PS was estimated using a multivariable logistic regression model. The dependent variable was the age of the patient, and the independent variables were all the variables that may confound the treatment-outcome relationship. The matching weights^16^ were then computed to create a pseudo-population (the weighted cohort) in which the distribution of baseline covariates is independent of the age of the patient.^12^ We used absolute standardized differences (ASDs) to assess the comparability of baseline covariates between the 2 groups. ASDs values close to 0 indicate insignificant differences between the groups. We consider that covariates with ASDs < 0.1 denotes unmeaningful imbalance (Online Supplemental Figure 1).^17^ Regression models were then used to compare all the outcomes in the weighted cohort. For binary outcomes, we performed univariate weighted log-binomial regression models. The presence of LVZs in the LA and its extent and the presence of LVZs in each atrial region were compared using the weighted Poisson regression model. Results are presented as relative risks with their 95% confidence intervals. *P* values <.05 were considered statistically significant. All the analyses were performed using R software version 4.1.1. (R Core Team, 2021, R Foundation for Statistical Computing, Vienna, Austria). Patient clinical characteristics were recorded (Online Supplemental Table 1).

### Electroanatomic voltage mapping

CA procedures were performed under general anesthesia using a 3-dimensional electroanatomic mapping system (CARTO 3, Biosense Webster, Diamond Bar, CA). A multipolar mapping catheter PentaRay (Biosense Webster) was used to create an LA voltage map of high density in SR at the procedure’s beginning. For patients in AF, electrical cardioversion was achieved to restore SR first.

LA was divided into 6 anatomical regions: anterior, septal, posterior, lateral, LA appendage (LAA), and inferior. The roof was part of the anterior region as previously described^9^ (Online Supplemental Figure 2).

The bipolar voltage amplitude was recorded for every point taken during LA mapping. The median LA (=all regions) bipolar voltage measurement was calculated. The LA intracavitary volume excluding the LAA was obtained for each patient after LA anatomic reconstruction and expressed in milliliters. The LA intracavitary volume index corresponded to the LA intracavitary volume indexed to the body surface and expressed in milliliters per square meter. A mapping LVZ was identified as an area of at least 1 cm^2^ with a bipolar peak-to-peak voltage amplitude of <0.5 mV.^18^ Each atrial region involving a LVZ was considered as a low-voltage region. The LVZs extent in the LA was categorized in each patient as stage I (no or discrete LVZ, ≤5%), II (mild LVZ, >5%–≤20%), III (moderate LVZ, >20%–≤35%), and IV (severe LVZ, >35%) according to the Utah fibrosis classification on the basis of the degree of atrial fibrosis detected using late gadolinium enhancement magnetic resonance imaging.^19^ For each patient, the LVZs extent was semiquantitatively assessed by an experienced operator blinded to clinical data.

### Radiofrequency CA procedure

PVI was performed using a 4-mm irrigated contact-force ablation catheter (ThermoCool SmartTouch, Biosense Webster) to deliver point-by-point contiguous lesions with an interlesion distance of <6 mm, a target temperature of 43 °C, and an infusion rate of 17 mL/min. A contact force of 5–20 g was obtained at each site with a lesion tag size of 2 mm. PVI was performed with high-power, short-duration ablation (50 W with a target ablation index of 450–500 on the anterior segment and 40 W with a target ablation index of 350–400 on the posterior segment).

The ablation protocol was the same in the 2 groups. In the case of a normal LA voltage map, PVI alone was performed (for stage I patients), whereas additional LVZs-guided ablation (LVZs homogenization or isolation) in addition to PVI was done in patients with mild or moderate LVZ. Linear ablation across LVZs was performed when the LVZs ablation area could be considered as a critical isthmus site for potential macroreentrant tachycardia. At the end of CA, atrial burst pacing to refractoriness was conducted to try to induce any tachycardia. No additional ablation was performed in the case of induced AF.

### Postablation follow-up

AADs were continued in all patients during a blanking period of 3 months after CA. Patients were seen at 3 months and then every 6 months until the 42nd month by their cardiologist. A 12-lead electrocardiogram and 24-hour Holter electrocardiogram were recorded to detect any atrial arrhythmia (AA) recurrence deﬁned by any documented AF, atrial flutter, and atrial tachycardia lasting >30 seconds. AADs were gradually discontinued between 3 and 6 months postablation in the absence of recurrence at the physician’s discretion. All this information was retrospectively obtained from their cardiologists by collecting consultation letters and various complementary examination results via phone and email.

### Statistical analysis

Quantitative variables with a non-normally distribution are presented as medians and interquartile ranges. Normally distributed variables are expressed as means with SDs. Differences in quantitative variables were evaluated for statistical significance using the Student *t* test or the Wilcoxon test, depending on data distribution. Categorical variables are given as numbers and percentages. Statistical differences in categorical variables between the 2 groups were tested using the χ^2^ test or Fisher exact test.

Kaplan-Meier survival curves were applied in each group to analyze freedom from AA recurrence after a single procedure. The log-rank test was used to compare the 2 groups.

Binominal logistic regression was used to calculate the odds ratio and a 95% confidence interval of independent variables associated with both LVZs and AF recurrences. Variables selected for testing in the multivariate analysis were those with *P*<.10 in the univariate analysis. All statistical analyses were performed using SPSS version 29.0 (IBM Corporation, Armonk, NY). A 2-tailed *P* value of <.05 was considered statistically significant.

## Results

### Baseline characteristics

Almost half of the patients ≥75 years of age were female (30 [45%] vs 74 [26%]; *P*<.01) with more cardiovascular risk factors. Those patients had a history of more paroxysmal AF (40 [60%] vs 106 [37%]; *P*<.01) and more sinus node dysfunction (11 [16%] vs 18 [6%]; *P*=.01) than did those <75 years of age. In patients ≥75 years of age, the P-wave duration was longer (160 [145–180] ms vs 139 [128–159] ms; *P*<.01) and renal function was lower (68 [50–78] mL/(min·1.73 m^2^) vs 88 [70–94] mL/(min·1.73 m^2^); *P*<.01). The LA intracavitary volume was significantly higher in patients >75 years of age (140 [128–164] mL vs 130 [110–160] mL; *P*<.01) and also its indexed values (74 [65–86] mL/m^2^ vs 62 [53–74] mL/m^2^; *P*<.01). The baseline characteristics before and after PS matching of the primary cohort are available in Table 1 and Online Supplemental Table 1, respectively.

**Table 1:** Baseline characteristics according to age

Characteristic	<75 y of age (n=286)	≥75 y of age (n=67)	*P*
Age (y)	63 (57–69)	76 (75–78)	<.01
Female sex	74 (26)	30 (45)	<.01
BMI (kg/m^2^)	30 (27–34)	28 (24–32)	.03
Dyslipidemia	110 (39)	38 (57)	<.01
Hypertension	177 (62)	55 (82)	<.01
Diabetes mellitus	54 (19)	9 (13)	.38
Smoking	38 (13)	3 (5)	.07
OSA	85 (30)	15 (22)	.29
CHA_2_DS_2_-VASc score	2 (1–3)	4 (3–4)	<.01
Paroxysmal AF history	106 (37)	40 (60)	<.01
Sinus node dysfunction	18 (6)	11 (16)	.01
Time to treatment∗ (d)	529 (224–1467)	880 (301–2216)	.07
Reported AF duration			.08
<3 mo	196 (69)	38 (57)	
≥3–<6 mo	35 (12)	10 (15)	
≥6–<9 mo	23 (8)	10 (15)	
≥9–<12 mo	10 (3.5)	6 (9)	
≥12 mo	21 (7.4)	3 (5)	
AF at the time of the procedure	101 (35)	24 (36)	>.99
β-Blocker	234 (82)	50 (75)	.24
Amiodarone	211 (74)	50 (75)	>.99
Flecainide	46 (16)	10 (15)	.96
Sotalol	3 (1)	2 (3)	.24
ACEi/ARB	170 (60)	47 (70)	.14
Aldosterone receptor antagonist	63 (22)	16 (24)	.87
P-wave duration (ms)	139 (128–159)	160 (145–180)	<.01
eGFR (mL/(min·1.73 m^2^))	88 (70–94)	68 (50–78)	<.01
LVEF (%)	60 (52–65)	60 (55–65)	.17
Per-procedural LAIV excluding LAA (mL)	130 (110–160)	140 (128–164)	<.01
Per-procedural LAIVI excluding LAA (mL/m^2^)	62 (53–74)	74 (65–86)	<.01

Data are presented as count (percentage) for categorical variables and as median (interquartile range) for quantitative variables. A 2-tailed *P* value of <.05 was considered significant.

ACEi/ARB = angiotensin-converting enzyme inhibitor/angiotensin II receptor blocker; AF = atrial fibrillation; BMI = body mass index; eGFR = estimated glomerular filtration rate; LAA = left atrial appendage; LAIV = left atrial intracavitary volume; LAIVI = left atrial intracavitary volume index; LVEF = left ventricular ejection fraction; OSA = obstructive sleep apnea.

∗ Time to treatment = time from the first clinical diagnosis of AF to the ablation procedure.

### LA bipolar voltage and LVZ assessment

In the unmatched cohort, the global LA bipolar voltage amplitude was lower in patients ≥75 years of age (1.5 [1.2–2.3] mV vs 2.4 [1.7–2.8] mV; *P*<.01) (Table 2).

**Table 2:** LA bipolar voltage amplitude and LVZ extent and distribution according to age

Variable	<75 y of age (n=286)	≥75 y of age (n=67)	*P*
LA bipolar voltage (mV)	2.4 (1.7–2.8)	1.5 (1.2–2.3)	<.01
No or discrete LVZ	200 (70)	22 (33)	<.01
Mild to severe LVZ	86 (30)	45 (67)	<.01
	*LVZ extent in the LA*		
Mild LVZ	54 (19)	25 (37)	<.01
Moderate LVZ	19 (6.7)	12 (18)	<.01
Severe LVZ	13 (4.6)	8 (12)	.08
	*Number of regional LVZs*		
Anterior	74 (26)	42 (63)	<.01
Septal	45 (16)	26 (39)	<.01
Posterior	29 (10)	19 (28)	<.01
Inferior	9 (3)	4 (6)	.28
Lateral	4 (1)	1 (1)	>.99
LAA	7 (2)	11 (16)	<.01

Data are presented as count (percentage) for categorical variables and as median (25th–75th percentile) for quantitative variables. A 2-tailed *P* value of <.05 was considered significant.

LA = left atrial; LAA = left atrial appendage; LVZ= low-voltage zone.

In the unmatched cohort, LVZs were found in 37% of the whole cohort (Table 2). Mild to severe LVZs were more frequent in patients ≥75 years of age (45 [67%] vs 86 [30%]; *P*<.01), especially in the case of mild LVZs (25 [37%] vs 54 [19%]; *P*<.01) and moderate LVZs (12 [18%] vs 19 [6.7%]; *P*<.01) (Table 2). When analyzing LVZs according to atrial region, patients ≥75 years of age had more anterior LVZs (42 [63%] vs 74 [26%]; *P*<.01), septal LVZs (26 [39%] vs 45 [16%]; *P*<.01), posterior LVZs (19 [28%] vs 29 [10%]; *P*<.01), and LVZs in the LAA (11 [16%] vs 7 [2%]; *P*<.01) than did patients <75 years of age (Table 2).

**Figure 2:**
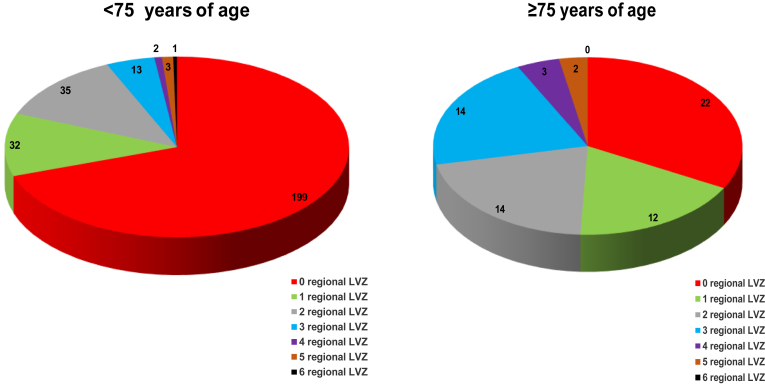
Pie charts showing the number of regional LVZs according to age. Patients <75 years of age had significantly fewer LVZs than did those >75 years of age (199 [70%] vs 22 [33%]; *P* < .01). We observed that 3 atrial regions with LVZ were significantly higher in patients >75 years of age (14 [21%] vs 13 [4.6%]; *P* < .01). In addition, 4 atrial regions with LVZ were more present at the limit of significance in patients >75 years of age (3 [4.5%] vs 2 [0.7%]; *P* = .05). LVZ = low-voltage zone.

When analyzing the number of regional LVZs according to age, younger patients had significantly fewer LVZs than did elderly patients (199 [70%] vs 22 [33%]; *P*<.01). Three atrial regions with LVZs were significantly higher in elderly patients (14 [21%] vs 13 [4.6%]; *P*<.01). In addition, 4 atrial regions with LVZs were more frequently present at the limit of significance in elderly patients (3 [4.5%] vs 2 [0.7%]; *P*=.05) (Figure 2).

After PS matching, mild to severe LVZs were more frequent in elderly patients (36.5 [64.9%] vs 29.9 [55.6%]; *P*=.226) without reaching the significance (Online Supplemental Table 2). However, after PS matching, mild LVZs were more frequently evidenced in elderly patients (*P*=.037) (Online Supplemental Table 2).

### Ablation results in the unmatched cohort

The procedure time (135 [112–157] minutes vs 126 [104–145] minutes; *P*=.02) and fluoroscopy time (27.4 [19.9–35.4] minutes vs 22 [17–30] minutes; *P*<.01) were longer in patients >75 years of age (Table 3).

**Table 3:** Procedural data–related characteristics

Variable	<75 y of age (n=286)	≥75 y of age (n=67)	*P*
Fluoroscopy time (min)	22 (17–30)	27.4 (19.9–35.4)	<.01
Procedure time (min)	126 (104–145)	135 (112–157)	.02
*Total mapping points taken per patient*			
4 LVZ subgroups	1521 (1156–1952)	1460 (1227–2300)	.64
No or discrete LVZ subgroup	1484 (1163–1741)	1428 (1220–1648)	.69
Mild LVZ subgroup	1568 (1118–1990)	1602 (1368–1730)	.48
Moderate LVZ subgroup	1460 (1120–1742)	1356 (1285–1436)	.46
Severe LVZ subgroup	1555 (1248–1973)	1450 (1384–1605)	.93
PVI alone	199 (70)	22 (33)	<.01
LVZs ablation	86 (30)	45 (67)	<.01
Linear ablation	42 (50)	29 (66)	.13
Anterior line	20 (48)	15 (52)	.92
Posterior line	25 (60)	17 (59)	>.99
Roof line	27 (65)	20 (69)	.88
Septal line	1 (2.4)	6 (21)	.02
Mitral line	7 (17)	6 (21)	.91
Complications	12 (4.5)	1 (1.5)	0.48
Scarpa’s hematoma	8 (2.8)	0 (0)	0.36
Stroke	1 (0.35)	0 (0)	>.99
Cardiac tamponade	1 (0.35)	0 (0)	>.99
Right phrenic paralysis	2 (0.7)	0 (0)	>.99

Data are presented as count (with percentage) for categorical variables and as median (25th–75th percentile) for quantitative variables. A 2-tailed *P* value of <.05 was considered significant.

LVZs = low-voltage zones; PVI = pulmonary vein isolation.

PVI alone was performed in 221 patients (62.6%) of the overall cohort, while the remaining 132 patients (37.4%) had additional LVZs-guided ablation.

PVI alone was more frequently performed in younger patients than in elderly patients (199 [70%] vs 22 [33%]; *P*<.01).

There was no difference between the 2 groups for linear ablation (29 [66%] vs 42 [50%]; *P*=.13) except for the septal line for elderly patients (Table 3).

Postprocedural complications occurred in 3.7% of the overall cohort (13 of 353), mainly perivascular complication. No difference was observed between the 2 groups (Table 3).

### Clinical outcome after persistent AF CA

**Figure 3:**
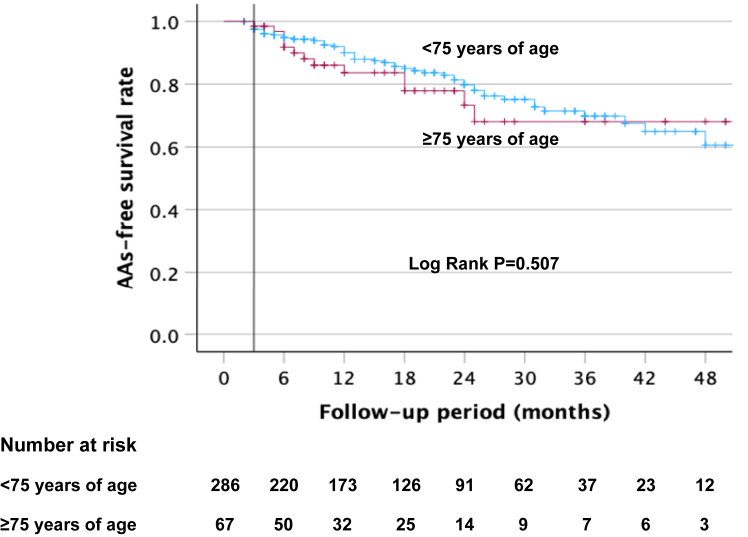
Kaplan-Meier survival curves showing the cumulative AF/AT recurrence-free survival rates according to age after a single procedure. AA = atrial arrhythmia; AF = atrial fibrillation; AT = atrial tachycardia.

After a follow-up period of 48.3 months (44.9–51.7 months) in this unmatched cohort, AA recurrence was observed in 65 of 353 patients (18.4%) after a single procedure. The Kaplan-Meier survival curves are shown in Figure 3. We observed no difference in AA-free survival rate after 1 procedure between the 2 groups (log-rank, *P* = .507).

After 12 months, 83.7%±5.1% of patients ≥75 years of age and 90%±2% of patients <75 years of age remained free of AF/atrial tachycardia (Figure 3). At 36 months, 68.1%±8.4% of patients ≥75 years of age and 69.7%±4.1% of patients <75 years of age were free of AAs. In the whole cohort, AADs were discontinued in 73.9% of patients (261 of 353).

Among patients without AA recurrence at 36 months, AADs were discontinued in 74.8% of the cohort (229 of 306): 73.2% in elderly patients (41 of 56) and 75.2% in younger patients (188 of 250) (*P*=.890).

The arrhythmia-free survival rate off AAD at 1-year follow-up was 88.4% and 92.3% in the elderly and younger groups, respectively (log-rank, *P*=.932). The arrhythmia-free survival rate off AAD at 2-year follow-up was 77.9% and 79.7% in the elderly and younger groups, respectively, after a single procedure (log rank, *P*=.932).

### Predictors of LVZ

To evaluate the predictors of LVZ in this unmatched cohort, univariate and multivariate analyses were performed in the whole cohort.

Female sex (*P*<.001), age ≥ 75 years (*P*=.042), renal function (*P*=.009), and LA volume index (LAVI) on the computer tomography (CT) scan (*P*<.001) were identified as predictors of LVZ in the global population of patients with persistent AF (Table 4).

**Table 4:** Predictors of LVZ in the global population of patients with persistent AF

Variable	Univariate analysis			Multivariate analysis		
	OR	95% CI	*P*	OR	95% CI	*P*
Female sex	5.547	3.381–9.099	<.001	4.683	2.550–8.602	<.001
Age ≥ 75 y	4.733	2.679–8.362	<.001	2.129	1.026–4.416	.042
AF duration > 6 mo	1.353	0.799–2.292	.260			
Time to treatment∗	1.000	1.000–1.000	.187			
BMI	0.975	0.939–1.013	.198			
Hypertension	2.673	1.628–4.388	<.001	1.816	0.976–3.378	.060
Diabetes mellitus	1.564	0.901–2.713	.112			
OSA	0.930	0.574–1.504	.766			
Coronary artery disease	1.193	0.644–2.208	.574			
Sinus node dysfunction	1.912	0.891–4.101	.096	0.905	0.339–2.415	.841
eGFR	0.960	0.948–0.973	<.001	0.978	0.962–0.994	.009
LAVI on the CT scan	1.054	1.039–1.070	<.001	1.054	1.036–1.072	<.001

Data are presented as an OR with 95% CI. A 2-tailed *P* value of <.05 was considered significant.

AF = atrial fibrillation; BMI = body mass index; CI = confidence interval; CT = computed tomography; eGFR = estimated glomerular filtration rate; LAVI = left atrial volume index; LVZ = low-voltage zone; OR = odds ratio; OSA = obstructive sleep apnea.

∗ Time to treatment = time from the first clinical diagnosis of AF to the ablation procedure.

### Predictive factors for AA recurrence

To evaluate the predictors of AA recurrence after CA in this unmatched cohort, univariate analysis was performed in the whole cohort. None of them were chosen as variables (Table 5).

**Table 5:** Predictors of AF recurrence after ablation in the global population

Variable	Univariate analysis			Multivariate analysis		
	OR	95% CI	*P*	OR	95% CI	*P*
Female sex	1.173	0.657–2.093	.589			
Age ≥ 75 y	1.079	0.549–2.121	.826			
AF duration > 6 mo	0.839	0.422–1.668	.616			
Time to treatment∗	1.000	1.000–1.000	.738			
BMI	0.975	0.929–1.024	.319			
Hypertension	0.586	0.338–1.013	.056			
Diabetes mellitus	0.803	0.384–1.677	.559			
OSA	1.520	0.858–2.694	.151			
Coronary artery disease	0.992	0.455–2.163	.985			
Sinus node dysfunction	1.778	0.750–4.214	.191			
eGFR	1.009	0.994–1.024	.250			
LAVI on the CT scan	1.005	0.991–1.020	.457			
CHA_2_DS_2_-VASc score	0.907	0.756–1.089	.296			
LVZs presence	1.453	0.842–2.508	.179			

Data are presented as an OR with 95% CI. A 2-tailed *P* value of <.05 was considered significant.

AF = atrial fibrillation; BMI = body mass index; CI = confidence interval; CT = computed tomography; eGFR = estimated glomerular filtration rate; LAVI = left atrial volume index; LVZs = low-voltage zones; OR = odds ratio; OSA = obstructive sleep apnea.

∗ Time to treatment = time from the first clinical diagnosis of AF to the ablation procedure.

## Discussion

### Main findings

In the present study, we report that in a unmatched cohort of patients with persistent AF, elderly patients display more LA electrophysiological remodeling with lower bipolar voltage, more LVZs, and higher LA volume. After matching the cohort according to cardiovascular risk factors, these differences are not so clear, testifying to the complex interplay of all cardiovascular risk factors in addition to age in the occurrence of LA remodeling.

Despite this significant fibrotic remodeling, patients had a similar and favorable 36-month outcome after a single voltage-guided ablation procedure with low complication rates. Female sex, age ≥ 75 years, renal function, and LA indexed volume were predictors of LVZ in patients with persistent AF. No predictive factors for AA recurrence could be identified in the whole cohort.

### LVZs extent and its regional distribution in elderly patients

Data on the LVZs extent and its regional distribution in elderly patients are currently scarce.^10^ Chen et al^20^ reported that the LVZ prevalence was as high as 41.4% in a population of older patients with paroxysmal AF. In our analysis, we evidenced that LVZs were even more present in 67% of elderly patients with persistent AF vs 30% of younger patients. We provided for the first time an information on the regional distribution and extent of the LVZs in elderly patients with persistent AF. Consistent with previous findings,^21^ we demonstrated that mild to severe LVZs were more frequent in elderly patients, especially mild and moderate LVZs. Consistent with previous findings,^9^ LVZs were more frequently localized in the septal, anterior, and posterior walls in both groups but more significantly in elderly patients and such a heterogeneous distribution may be explained, at least in part, by LA regional differences in wall stress.^22^

When analyzing LVZs by atrial region, patients with 3 low-voltage atrial regions of the LA were 3 times more common in elderly patients.

### Favoring factors for LVZ

Conflicting data on the potential association between age and LVZ are published, with several studies confirming this association^8^^,^^9^ whereas other studies showed the opposite.^23^^,^^24^ We evidenced using a multivariate analysis in the whole homogeneous cohort of patients with persistent AF that age ≥ 75 years was a predictor of LVZ.

Nevertheless, to minimize confounding factors within the 2 groups, we have used PS matching. After PS matching, LVZs were more present in elderly patients without reaching the statistical significance. The limited number of patients in the elderly group might explain these results. However, it can be still observed that mild LVZs were more frequent in elderly patients.

These discrepancies can also be explained by the complex interplay between cardiovascular risk factors and age in the occurrence of LVZs. Indeed, aging is associated with a high number of comorbidities that increase with age. It is now well established that these comorbidities common in AF^25^^,^^26^ have all together a synergistic impact on AF occurrence, substrate remodeling, and the risk of stroke. In our study, one-third of patients with AF had at least 3 associated comorbidities.

Female sex was also a predictive factor for LVZ. In our cohort, almost half of the elderly patients were female. Several studies have already reported such an association.^8^^,^^24^ Hormonal changes and estrogen decrease could be involved in LA remodeling.^27^ Sex differences occurring in the genetic expression of certain protein pathways could also explain an increase in LA fibrosis in women.^28^ Some authors also incriminate the metabolism of epicardial fat to explain differences in inflammation and thus LA remodeling.^29^

Interestingly, renal dysfunction was predictive of LVZ occurrence. Despite not being an element of the CHAD_2_DS_2_-VASc score, renal dysfunction is associated with most of the items of the score. It favors AF, but AF itself contributes to increase renal dysfunction through decreased perfusion. Matsuda et al^30^ reported an association between renal dysfunction and LVZ presence in patients undergoing AF ablation with chronic renal disease, defined as estimated glomerular filtration rate < 60 mL/(min·1.73 m^2^). In their cohort of patients with paroxysmal and persistent AF, the calculated optimal cutoff value to predict LVZ was 71.5 mL/(min·1.73 m^2^). In patients with renal failure, LA pressure is increased. There is a sympathetic hyperactivity, an activation of the renin-angiotensin-aldosterone system, and an increase in oxidative stress—all these pathways contributing to atrial fibrosis activation.^31^

Similarly, LAVI on the CT scan was predictive of LVZ occurrence. LA size is certainly an important parameter of remodeling in persistent AF. Nevertheless, increased LA diameter was not always associated with LVZ.^32^ In our cohort, LAVI associated with age > 75 years could be more powerful in predicting remodeling. Several studies on AF ablation have reported an association between LA enlargement and the presence of LVZs on voltage maps.^8^^,^^33^

### LVZs-guided ablation results and the predictor of AA recurrence

The purpose of the study was to evaluate the safety and efficacy of AF CA in a cohort of elderly patients with persistent AF, particularly with a voltage-guided ablation strategy. Patients with advanced age are often excluded from randomized controlled trials, and data are limited in this subgroup of the population.

In 2016, Bunch et al^34^ observed that age significantly affected outcomes after AF ablation when analyzed with long-term follow-up, particularly in patients >70 years of age.

Metzner et al^35^ observed in their cohort of patients >75 years of age with paroxysmal and persistent AF that the safety profile was comparable with that in patients of younger age. On the contrary, the described results of CA were less promising in persistent and long-standing persistent AF. Our data showed a better maintenance of SR after the follow-up period. The technical approach in their study was different with targeting complex fractionated atrial electrograms after PVI and cardioversion in the case of reinitiation of AF after cardioversion or unsuccessful cardioversion. Contact force catheters and multipolar mapping catheters were not available at that time.

We could also observe in our cohort that the proportion of patients being in SR at the time of the procedure was quite important, around 35% both in the youngest group and in the oldest group. This is an important point in explaining the positive results of the ablation procedures and the absence of difference in the outcome between the 2 groups.

In our study, despite additional ablation, a low complication rate was reported. We could observe the same safety profile of AF CA with mainly minor complications related to perivascular complications.

Given the increasing proportion of elderly patient candidates for CA, recent studies have evaluated the results of rapid 1-shot systems in this group. Boehmer et al^5^ observed that elderly patients with persistent AF had a significant risk of symptomatic AF recurrence after cryo-PVI than did matched patients of younger age at 24 months (45% vs 18.8%, respectively).

Several studies have compared the outcomes of CA of AF in elderly patients and reported comparable efficacy of cryoballoon and radiofrequency ablation of AF in elderly patients.^36^^,^^37^ But the arrhythmia-free survival rate off AAD at 1-year follow-up was 47.3% and 55.4% in the radiofrequency and cryoballoon groups, respectively. LA remodeling and LVZ presence might explain less interesting results of 1-shot system ablation in the persistent AF elderly subgroup. With a LVZs-guided ablation associated with PVI, a tailored AF ablation, we report favorable outcomes with no difference between the 2 groups of age, with 68% of elderly patients and 70% of younger patients free of AA recurrence at 36 months. Interestingly, the proportion of patients without AADs was quite important but similar in the 2 groups, around 73% in the elderly group and 75% in the younger group.

The results of the voltage-guided ablation strategy in addition to PVI are encouraging in elderly patients with persistent AF, but data are still scarce. Further randomized studies are needed to confirm these results. Chen et al^20^ recently reported that additional low-voltage area ablation beyond PVI decreased AF recurrence in older patients with paroxysmal AF compared with circumferential PVI alone in a cohort of patients aged 65–80 years.

We could not find any predictive factor for AA recurrence after a single procedure in the whole cohort. This lack of significant findings may be attributed to the small size of the cohort, which could have limited the statistical power to detect meaningful associations. In our analysis, LVZ presence was not a predictor of AF recurrence after LVZ-guided ablation. This may be explained by the ablation strategy itself, which targets these LVZs in addition to PVI. The single-center retrospective observational study with a relatively small sample size and variability in AAD discontinuation could also be another explanation. For these reasons, results have to be carefully interpreted.

### Study limitations

This study has several important limitations. First, it is a single-center study with an observational long-term analysis. The number of patients in this retrospective analysis is limited, and larger patient populations are needed. This limits the strengths of the conclusions, as the matched groups are subject to confounding variables that can influence the results concerning LVZs. Therefore, we have used PS matching to minimize confounding factors in the occurrence of LVZ in 2 groups. The CA results were assessed until 36 months postablation. Outcomes after a longer follow-up period would be of interest for the repeat procedure assessment. In addition, the discontinuation of AADs could not be obtained for the whole cohort because follow-up was performed by the patients’ individual cardiologists. It could have influenced the results of ablation.

## Conclusion

This study reports increased LA substrate remodeling with lower bipolar voltage, more frequent LVZs, and larger LA volume in a cohort of patients >75 years of age with persistent AF compared with younger patients. Analyzing the matched cohorts, these differences were no longer observed, possibly because of the size of the population and the complex interplays linking cardiovascular risk factors, age, and LA remodeling. Age ≥ 75 years, female sex, renal function, and LA indexed volume measured using the CT scan were all independent predictors of LVZ presence.

LA scar did not seem to negatively affect the outcome after a single voltage-guided substrate persistent AF ablation procedure, and the rate of complications was low. Further studies are needed to confirm these observations. Age should not limit the realization of persistent AF ablation in symptomatic elderly patients.

## Funding Sources

This work was supported by GERCA (Groupe pour l’Enseignement et la Recherche Cardiovasculaire en Alsace).
