# Supplementary material for: Left atrial remodeling and voltage-guided ablation outcome in persistent atrial fibrillation patients over 75 years of age
Source: Heart Rhythm O2. 2024 Dec 24;6(3):307–16. doi: 10.1016/j.hroo.2024.12.006 (PMC11973668; doi:10.1016/j.hroo.2024.12.006)
Supplement: Supplemental Tables and Figures [file mmc1.doc]

**Table S1.** Baseline characteristics according to age before and after PS-matching.

|  |  | | **Before PS-matching** | | | | **PS-matched** | | | |
| --- | --- | --- | --- | --- | --- | --- | --- | --- | --- | --- |
|  | | **Total**  **(n=353)** | | **<75 years of age (n=286)** | **≥75 years of age (n=67)** | **ASD** | **<75 years of age (n=53,8)** | **≥75 years of age (n=56,3)** | | **ASD** |
| Female gender | | 104 (29,5) | | 74 (25,9) | 30 (44,8) | **0,403** | 23,0 (42,7) | 23,8 (42,3) | 0,009 | |
| AF duration > 6 months, n (%) | | 73 (20,7) | | 54 (18,9) | 19 (28,4) | **0,223** | 13,8 (25,7) | 15,5 (27,5) | 0,040 | |
| Time to treatment, days | | 577 [234-1595] | | 529 [224-1467] | 880 [301-2126] | **0,264** | 1211 [382-2341] | 829 [287-2158] | 0,023 | |
| BMI, kg/m² | | 29,5 [26-33] | | 30 [27-33,8] | 28,4 [24,2-32] | **0,325** | 28,3 [26-32] | 29 [24,5-32] | 0,030 | |
| Dyslipidemia, n (%) | | 148 (41,9) | | 110 (38,5) | 38 (56,7) | **0,372** | 27,0 (50,2) | 29,9 (53,2) | 0,059 | |
| Hypertension, n (%) | | 232 (65,7) | | 177 (61,9) | 55 (82,1) | **0,462** | 42,8 (79,5) | 45 (79,9) | 0,010 | |
| Paroxysmal AF history, n (%) | | 146 (41,4) | | 106 (37,1) | 40 (59,7) | **0,465** | 31,4 (58,3) | 32,5 (57,7) | 0,012 | |
| Sinus node dysfunction, n (%) | | 29 (8,2%) | | 18 (6,3%) | 11 (16,4%) | **0,323** | 6,6 (12,2) | 7,4 (13,2) | 0,030 | |
| eGFR, mL/min/1,73² | | 82 [66-92] | | 87,5 [70-94] | 68 [50-78] | **0,915** | 70 [54-88] | 69 [57-80] | 0,018 | |
| Per-procedural LAIVI excluding LAA, ml.m^2^ | | 64 [55-77] | | 62 [53-74] | 74 [65-86] | **0,668** | 70,53 [60-83] | 72 [62-84] | 0,038 | |
| P-wave duration, ms | | 140 [128-160] | | 139 [128-159] | 160 [144,5-180] | **0,795** | 160 [138-177] | 159 [143-180] | 0,037 | |

Data are presented as a value (with percentage) for categorical variables, median (25th-75th percentile) for quantitative variables.

ASD values close to 0 indicate insignificant differences between groups. We consider that covariates with ASD < 0.1 denotes unmeaningful imbalance.

Time to treatment =time from first clinical diagnosis of AF to ablation procedure.

**Abbreviations:** *PS, Propensity score; ASD, Absolute standardized differences ; BMI, Body Mass Index; OSA, AF, Atrial Fibrillation; eGFR, Estimated Glomerular Filtration rate; LAA, Left Atrial Appendage; LAIVI, Left Atrial Intracavitary Volume Index.*

**Table S2.** Low-Voltage Zones (LVZs) extent and distribution according to age after PS-matching.

| **Variable** | **Age < 75 years old**  **(n = 53.8)** | **Age ≥ 75 years old**  **(n = 56.3)** | **RR [95%CI]** | **P value** |
| --- | --- | --- | --- | --- |
| No or discret LVZ, n (%) | 23.9 (44.4) | 19.8 (35.1) | 0.79 [0.53 – 1.18] | 0.256 |
| Mild to severe LVZ, n (%) | 29.9 (55.6) | 36.5 (64.9) | 1.17 [0.91 – 1.49] | 0.226 |
| Mild LVZ, n (%) | 13.1 (24.4) | 22.5 (40.0) | 1.64 [1.03 – 2.60] | **0.037** |
| Moderate LVZ, n (%) | 9.6 (17.9) | 8.7 (15.5) | 0.87 [0.41 – 1.81] | 0.704 |
| Severe LVZ, n (%) | 7.1 (13.3) | 4.6 ( 8.1) | 0.61 [0.23 – 1.64] | 0.330 |
| Anterior LVZ, n (%) | 27.2 (50.7) | 33.5 (59.5) | 1.18 [0.89 – 1.56] | 0.260 |
| Septal LVZ, n (%) | 27.2 (50.7) | 20.6 (36.6) | 1.11 [0.71 – 1.72] | 0.644 |
| Posterior LVZ, n (%) | 13.7 (25.5) | 13.8 (24.6) | 0.97 [0.55 – 1.70] | 0.904 |
| Inferior LVZ, n (%) | 3.3 ( 6.1) | 2.0 ( 3.5) | 0.58 [0.16 – 2.14] | 0.410 |
| Lateral LVZ, n (%) | 1.7 ( 3.1) | 0.6 ( 1.1) | 0.36 [0.037 – 3.49] | 0.378 |
| LAA LVZ, n (%) | 4.1 ( 7.7) | 8.2 (14.6) | 1.91 [0.67 – 5.42] | 0.228 |
| Number of regional LVZ in LA | 1.26 ± 1.43 | 1.42 ± 1.34 | 1.12 [0.81 – 1.56] | 0.492 |
| ≥3 low-volted regions in LA, n(%) | 9.4 (17.6) | 12.9 (23.0) | 1.31 [0.68 – 2.53] | 0.428 |

All data are presented as a value (percentage) for categorical variables or mean±SD for quantitative variables. A two-tailed p value<0.05 was considered significant.

**Abbreviations:** *PS, Propensity score, LVZ, Low voltage zone; RR, Relative risk; CI, confidence interval; LAA, left atrial appendage ; LA, left atrium.*


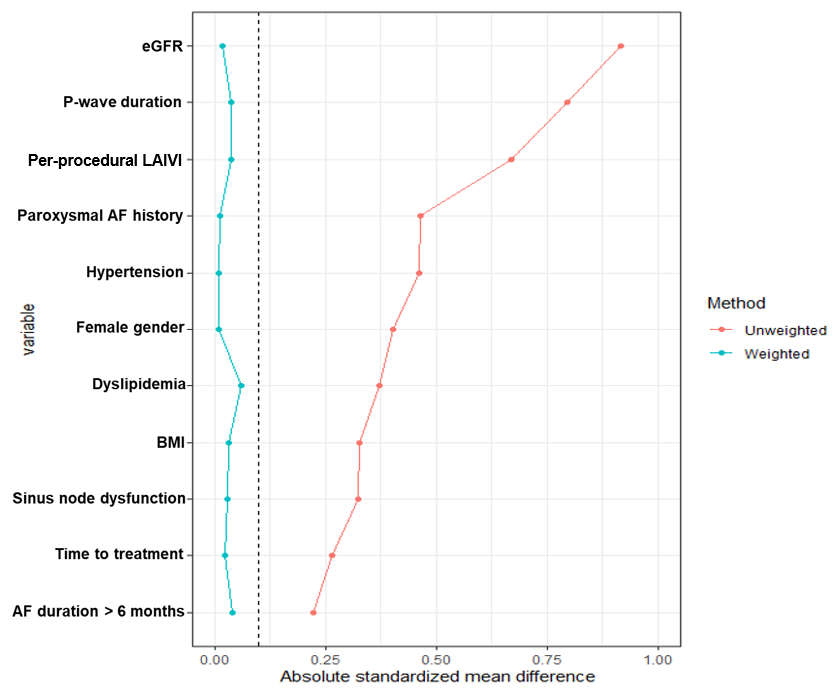


**Figure S1.** Mean absolute standardized differences (ASD) of the different variables before and after weighting on the two groups.

Time to treatment =time from first clinical diagnosis of AF to ablation procedure. Absolute standardized differences (ASD) were used to determine the influence of covariates on the two groups. Groups are generally considered similar when the ASD is < 0,1.

**Abbreviations:** *ASD, Absolute standardized differences ; eGFR, Estimated Glomerular Filtration rate; LAIVI, Left Atrial Intracavitary Volume Index; AF, Atrial Fibrillation; BMI, Body Mass Index.*

*
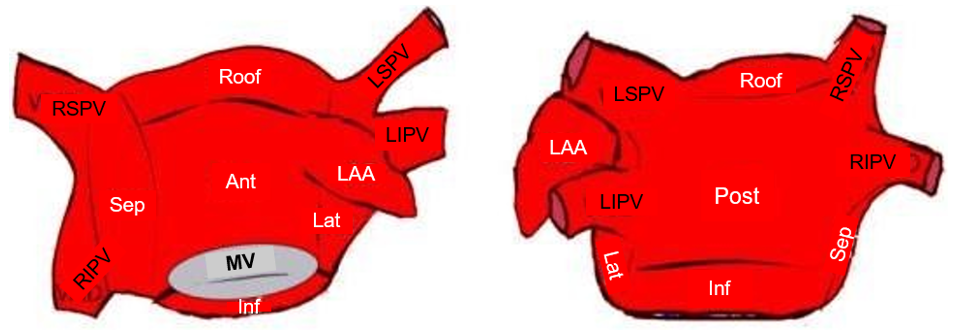
*

**Figure S2.** Anterior and posterior views of left atrium segmented into different regions.

The LA was divided into 6 regions : septum, LA appendage, anterior, posterior, inferior, and lateral walls. The roof was part of the anterior region.

**Abbreviations:** *LSPV, left superior pulmonary vein; LIPV, left inferior pulmonary vein; RIPV, right inferior pulmonary vein; RSPV, right superior pulmonary vein; Ant; anterior, Sep; septum, Lat, lateral, Inf; inferior, Post, posterior, LAA; left atrial appendage, MV; mitral valve.*
